# Suprascapular Lipoblastoma Extending in to the Thorax

**Published:** 2013-05-27

**Authors:** Federica Pederiva, Giulio Andrea Zanazzo, Massimo Gregori, Jurgen Schleef

**Affiliations:** Division of Pediatric Surgery, Institute for Maternal and Child Health - IRCCS “Burlo Garofolo” – Trieste, Italy; Division of Hematology-Oncology, Institute for Maternal and Child Health - IRCCS “Burlo Garofolo” – Trieste, Italy; Division of Radiology, Institute for Maternal and Child Health - IRCCS “Burlo Garofolo” – Trieste, Italy; Division of Pediatric Surgery, Institute for Maternal and Child Health - IRCCS “Burlo Garofolo” – Trieste, Italy

**Keywords:** Thoracoscopy, Thoracic tumor, Lipoblastoma

## Abstract

Lipoblastoma is a rare benign soft-tissue neoplasm that occurs most commonly in children less than 3 year of age. We present a case of left suprascapular lipoblastoma in an 11-month-old boy which grew into the thorax and was approached by thoracoscopy. In this case thoracoscopic approach was the best option to reach the intrathoracic component of the mass in the apex of the left side of the chest.

## INTRODUCTION

Lipoblastoma is a rare soft tissue tumor occurring predominantly in infancy and early childhood [1, 2]. Most lipoblastomas arise within the soft tissue of the trunk and the extremities, but they may also occur in the head, neck, mediastinum, retroperitoneum, lungs, heart, and salivary glands [2]. They commonly present as a painless mass that can grow variably [3]. Despite their benign biological behavior they may become symptomatic by compressing adjacent organs and tissues. Complete surgical excision is the best therapeutic option to prevent recurrences [4]. We present a case of suprascapular lipoblastoma which was extending into the thorax.

## CASE REPORT

An 11-month-old boy was referred with a growing left suprascapular mass without any other accompanying symptoms. The patient had no history of trauma. On physical examination, a 3 cm x 3 cm firm, painless mass was palpated. It was well circumscribed with normal overlying skin. Results of laboratory tests tumor markers (α-fetoprotein, serum β human chorionic gonadotrophin, carcinoembryonic antigen and urinary catecholamine metabolites) were within normal limits. An ultrasound color Doppler detected a heterogeneous mass with a hyperechoic component extending into deeper tisues with poorly defined limits. Computed tomographic (CT) chest scan revealed a fatty tumor with well-defined septae and focal densities corresponding to myxoid regions. Magnetic resonance imaging (MRI) showed a well-circumscribed mass arising from suprascapular soft tissues, having a signal consistent with adipose tissue and growing through the ribs into the left chest cavity (Fig. 1). A biopsy of the mass in the left suprascapular region was performed to make a definitive diagnosis. The extra-thoracic component of the mass did not invade the surrounding tissues. Histopathological examination revealed a lipoblastoma, with a characteristic lobular architecture and fibrous septa between nodules of adipose tissue in various stages of maturation.

A combined surgery was scheduled performing left thoracoscopy and open surgery through the previous suprascapular incision to completely excise the intrathoracic and extrathoracic components of the mass. The patient was positioned semi-prone with the left side slightly elevated. A 5 mm port for the videoscope was placed one intercostal space below the tip of the scapula, followed by three 3 mm working ports placed under direct vision in the 6th to 8th intercostal space paravertebrally in the anterior axillary line. Targeting an intrathoracic pressure of 3 to 5 mmHg, CO2 insufflation was initiated with 1 to 2 L/min. The intrathoracic part of the mass in the upper chest cavity was excised (Fig. 2) and brought out through the suprascapular incision. The postoperative course was uneventful. Two years later, MRI revealed an enlarging extrathoracic soft tissue mass which was believed to be a local recurrence. A complete re-excision of the mass was carried out through the previous suprascapular incision. Histopathological examination showed fatty tissue and did not confirm the recurrence of lipoblastoma.

**Figure:**
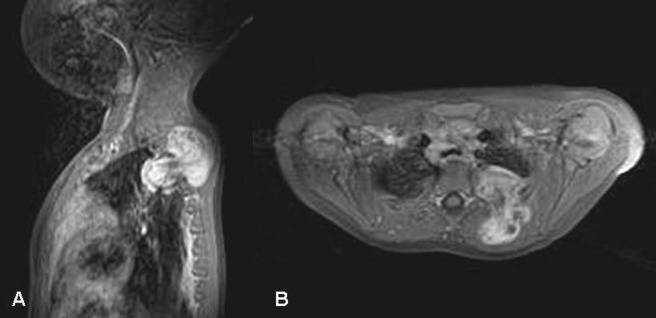
Figure 1: T1-weighted sagittal (A) and axial (B) MRI of the cervico-dorsal region showing a hyperintense well-circumscribed mass arising from suprascapular soft tissue and growing through the ribs into the left chest cavity.

**Figure:**
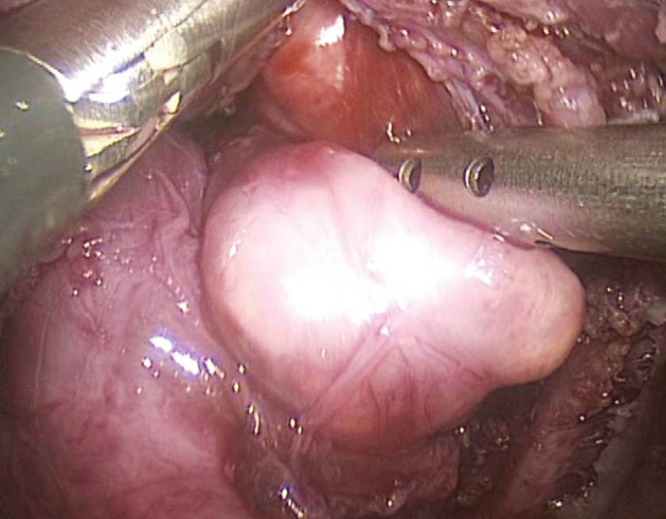
Figure 2: Intraoperative image showing the isolation of the intrathoracic part of the mass in the apex of the chest.

## DISCUSSION

Lipoblastoma is a rare, benign tumor of the embryonal fat occurring mostly in children and infants less than 3 years of age, with a male predominance.[1] Since its first description, two clinico-pathological entities have been defined, the superficially located, circumscribed benign lipoblastoma, clinically similar to lipoma, and the diffuse lipoblastomatosis, originating in deep soft tissues with an infiltrative pattern. It has recently been suggested that this distinction may not be clinically relevant, because both circumscribed and infiltrative lipoblastomas can recur.[3] Lipoblastoma may arise anywhere within the soft tissue with a predilection for the extremities and trunk, although more recent series have reported a lower incidence of extremity lipoblastomas. The most common presenting symptom is a painless mass increasing in size, as noted in our case, otherwise its rapid growth can cause compressive symptoms.[1] Although preoperative imaging may be useful to assess the extent of the disease that helps in planning surgical resection, it can not differentiate lipomatous tumors, which are radiologically indistinguishable.[5] Definitive diagnosis of the tumor is therefore made on histopathology as in our case.

A complete nonmutilating surgical excision remains the treatment of choice for lipoblastoma which has a propensity to enlarge and to cause cosmetic and functional consequences. If the entire tumor can not be safely removed at the time of initial resection, a staged approach is recommended. After complete resection the prognosis is excellent with the recurrence rate of less that 25%. Metastases are not reported. [4, 6] In our case, biopsy was a necessary step to clarify the preoperative diagnosis and to exclude a malignant process such as rhabdomyosarcoma or neuroblastoma. Once the pathologist had confirmed the benign nature of the lesion, a combined surgery was scheduled. Thoracoscopic approach was the best option to reach and excise the intrathoracic mass in the apex of the left side of the chest. Recent advances in minimally invasive surgery in children have allowed for a wide expansion of applications. The greatest advantage of a thoracoscopic approach is avoidance of a posterolateral thoracotomy and providing an excellent visualization of the anatomy even in hard to reach locations.[7] Even total gross excision was believed to be achieved, the follow up revealed an extrathoracic recurrence. A “wait and see” approach was initially adopted, but the concern for the progressive enlargement of the mass led to a second surgery. To conclude, lipoblastoma should be considered in the differential diagnosis of rapidly growing soft fatty masses of children within the thorax and the mediastinum.

## Footnotes

**Source of Support:** Nil

**Conflict of Interest:** None declared

